# DNA Damage and Inflammatory Response of p53 Null H358 Non-Small Cell Lung Cancer Cells to X-Ray Exposure Under Chronic Hypoxia

**DOI:** 10.3390/ijms252312590

**Published:** 2024-11-23

**Authors:** Hasan Nisar, Melanie Brauny, Frederik M. Labonté, Claudia Schmitz, Bikash Konda, Christine E. Hellweg

**Affiliations:** 1 Department of Radiation Biology, Institute of Aerospace Medicine, German Aerospace Center (DLR), 51147 Cologne, Germany; hasanisar@pieas.edu.pk (H.N.); bikash.konda@dlr.de (B.K.); 2 Department of Medical Sciences, Pakistan Institute of Engineering and Applied Sciences (PIEAS), Islamabad 44000, Pakistan; 3 Interfaculty Institute of Microbiology and Infection Medicine, Faculty of Science & Faculty of Medicine, University of Tübingen, 72074 Tübingen, Germany; 4 Department of Biology, Faculty of Mathematics and Natural Sciences, University of Cologne, 50923 Cologne, Germany

**Keywords:** ionizing radiation, hypoxia, lung cancer, survival, cell cycle, DNA double-strand breaks, DNA repair, interleukin expression, non-small cell lung cancer cells, radioresistance

## Abstract

Hypoxia-induced radioresistance limits therapeutic success in cancer. In addition, p53 mutations are widespread in tumors including non-small cell lung carcinomas (NSCLCs), and they might modify the radiation response of hypoxic tumor cells. We therefore analyzed the DNA damage and inflammatory response in chronically hypoxic (1% O_2_, 48 h) p53 null H358 NSCLC cells after X-ray exposure. We used the colony-forming ability assay to determine cell survival, γH2AX immunofluorescence microscopy to quantify DNA double-strand breaks (DSBs), flow cytometry of DAPI-stained cells to measure cell cycle distribution, ELISAs to quantify IL-6 and IL-8 secretion in cell culture supernatants, and RNA sequencing to determine gene expression. Chronic hypoxia increased the colony-forming ability and radioresistance of H358 cells. It did not affect the formation or resolution of X-ray-induced DSBs. It reduced the fraction of cells undergoing G2 arrest after X-ray exposure and delayed the onset of G2 arrest. Hypoxia led to an earlier enhancement in cytokines secretion rate after X-irradiation compared to normoxic controls. Gene expression changes were most pronounced after the combined exposure to hypoxia and X-rays and pertained to senescence and different cell death pathways. In conclusion, hypoxia-induced radioresistance is present despite the absence of functional p53. This resistance is related to differences in clonogenicity, cell cycle regulation, cytokine secretion, and gene expression under chronic hypoxia, but not to differences in DNA DSB repair kinetics.

## 1. Introduction

The treatment of lung cancer remains an area of rigorous research since it remains the leading cause of cancer-related mortality [1]. Almost 80% of lung cancers are non-small cell lung carcinomas (NSCLCs) [2,3]. Radiotherapy continues to be integral to a multimodality approach to treat NSCLCs [4]. Efforts to improve radiotherapy outcomes through modifications in dose fractionation and dose delivery techniques, the synergistic targeting of the immune system, and the addition of radiosensitizers have been utilized to achieve a limited improvement in the overall survival [4,5,6,7,8,9,10]. Moreover, a rigorous exploration of the biological mechanisms governing radioresistance in NSCLCs is underway [11,12,13,14,15,16].

An important cause of radioresistance in solid tumors including NSCLCs is tumor cell hypoxia [17,18,19]. Hypoxic regions in NSCLC tumors in vivo have been confirmed through multiple positron emission tomography (PET) studies using radiotracers specific for hypoxia [20,21,22]. Several clinical studies have demonstrated tumor hypoxia to be associated with the poor prognosis of NSCLCs [21,23,24]. The median molecular oxygen (O_2_) concentration in healthy lung tissue is 5.6%, about threefold higher than the median O_2_ concentration observed in lung tumors (~1.9%) [25]. Such lower cellular concentration of O_2_ can reduce the intended cytotoxicity of ionizing radiation in radiotherapy. The physical explanation of this phenomenon is generally based on a lower fixation of reactive oxygen species (ROS); both in normal and in cancerous tissues, energy deposited by ionizing radiation such as X-rays causes the radiolysis of water. This leads to the generation of primary radicals, such as reducing hydrogen radicals (H^•^), hydroxyl radicals (OH^•^) and hydrated electrons (e^−^_aq_), which can react with O_2_ to superoxide anion (O_2_^•−^) and hydroxyl-peroxyl radical (HO_2_^•^). All radicals in turn may lead to DNA damage. Finally, hydrogen peroxide (H_2_O_2_) which has a longer lifetime and diffusion range, can be formed by the reaction of two OH^•^ or H^•^ and HO_2_^•^, and inflict DNA damage further away from the primary ionizations via the Fenton reaction. The inflicted DNA damage can encompass DNA double-strand breaks (DSBs). Failure to repair DNA DSBs leads to cell death [19]. Lack of O_2_ at the time of irradiation, therefore, may lead to lower ROS-induced cytotoxicity. Additionally, several cellular adaptations to chronic hypoxia in NSCLC cell lines and others have been reported that alter cell metabolism, motility, migration, proliferation, and inflammatory response. These cellular adaptations are more likely to arise in response to chronic hypoxia rather than acute hypoxia and may enhance tumor cell survival against treatment options such as irradiation [5,26,27,28,29].

The therapeutic targeting of hypoxia to improve tumor control in NSCLCs has not as yet led to any well-documented survival advantages. This indicates a need for further investigating the cellular response to ionizing radiation in chronically hypoxic NSCLC cells [24,26,30]. The advent of hypoxia workstations such as the one used in this work may aid this cause by allowing for controlled and continuous or intermittent hypoxic conditions to now be maintained in vitro for the more accurate simulation of in vivo tumor hypoxia.

Classically, the cellular DNA damage response (DDR) to ionizing radiation comprises cell cycle arrest alongside DNA damage repair and the activation of cell death pathways to remove cells with unrepairable damage; the p53 protein, encoded by the tumor protein p53 (TP53, or p53) gene, plays a pivotal role in the DDR in normal cells [31]. Not only is it essential for cell cycle arrest at the G1/S phases checkpoint but it is also important for cell cycle arrest at the G2/M checkpoint [32]. Key cell death pathways such as apoptosis, autophagy, and senescence are heavily reliant on the presence of a functioning p53 protein for their successful initiation and execution [33]. Additionally, p53 controls the transcription of the radioprotective glutathione peroxidase, which reduces H_2_O_2_ to water molecules while oxidizing glutathione to glutathione disulfide, as well as XPC and Ku80, which are important components of the nucleotide excision repair and non-homologous end-joining repair pathways, respectively [34].

However, *TP53* is one of the most mutated genes in human cancers. Furthermore, chronic hypoxia can exert selection pressure in favor of cancer cell clones with inactivated *TP53* genes since p53-proficient cells tend to undergo apoptosis in response to chronic hypoxia [35,36]. *TP53* mutations were observed in up to 50% of NSCLCs, making it the most mutated gene in NSCLC [37]. Most of these mutations lead to protein inactivation, affecting cell cycle regulation and cell death pathways [37,38]. Chronic hypoxia has also been reported to inhibit p53 transactivation, thereby enhancing cell survival in p53-wt cells but not in p53-null cells [39]. To the best of our knowledge, very limited data are available exploring the response of p53 null NSCLC cells to ionizing irradiation under hypoxia [40]. The DDR of chronically hypoxic p53 null NSCLC cells following irradiation may be impacted in terms of cell survival, cell cycle regulation, DSB induction or resolution, and inflammatory response.

**Figure 1:**
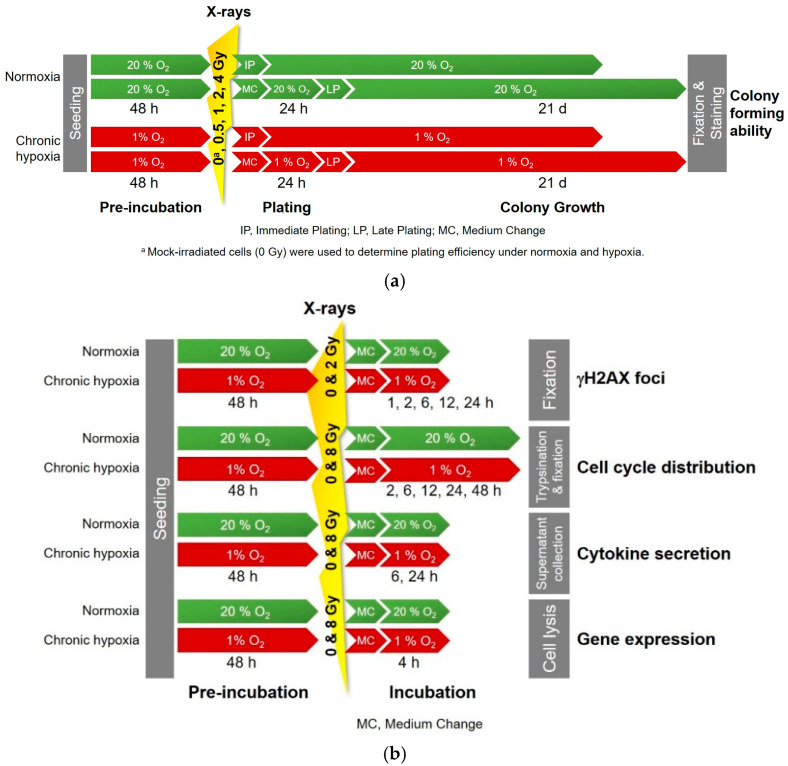
Timeline, oxygen, and irradiation conditions of experiments with H358 cells. Before irradiation, hypoxic cells were incubated with 1% O_2_ and normoxic cells with 20% O_2_ for 48 h. Oxygen conditions were kept during and after irradiation until the end of the experiment. (**a**) Colony forming ability tests; (**b**) experiments performed to determine cell cycle distribution, number of γH2AX foci indicating DNA double-strand break (DSB) induction and repair, secretion of the cytokines interleukin-6 and -8 (IL-6 and IL-8), and gene expression by RNA sequencing.

The purpose of this work was to shed more light on the possible cellular mechanisms playing a role in hypoxia-induced radioresistance in NSCLC. Therefore, we analyzed the response to X-ray exposure (0–8 Gy) of chronically hypoxic (48 h exposure to 1% O_2_) H358 cells—a p53-null human NSCLC cell line—compared to normoxic controls, considering cell survival, cell cycle progression, DNA double-strand break (DSB) induction and repair, and interleukin (IL-6 and IL-8) production (Figure 1a,b). Furthermore, any such differences were co-related with the differential gene expression in H358 cells following X-ray exposure under hypoxia and normoxia (Figure 1b).

## 2. Results

### 2.1. Chronic Hypoxia Enhances the Colony-Forming Ability of H358 Cells

The colony-forming ability of H358 and p53-wildtype A549 cells was determined as the plating efficiency after 48 h and 72 h incubation under either normoxia or hypoxia (see Section 4.3). The hypoxic cells were seeded and allowed time for colony growth while maintaining low oxygen concentration (1% O_2_), as opposed to their normoxic controls, which were seeded and allowed to grow and form colonies at 20% O_2_ levels. Overall, the plating efficiency of the two cell lines ranged from 16 to 31%, indicating that colonies can grow under hypoxia (Table 1).

The plating efficiency of H358 (p53-null) cells pre-incubated under 1% O_2_ for 72 h (0 Gy controls of the late plating CFA experiments) was statistically significantly enhanced in comparison to normoxic controls (Table 1). This was in contrast to A549 (p53-wt) cells, which exhibited a statistically significantly lower plating efficiency when compared to their respective normoxic controls.

Hypoxic pre-incubation for 48 h (0 Gy controls of the immediate plating CFA experiments) resulted in no significant difference in plating efficiency compared to normoxic controls.

### 2.2. Hypoxia Increases Radioresistance of H358 Cells

**Figure 2:**
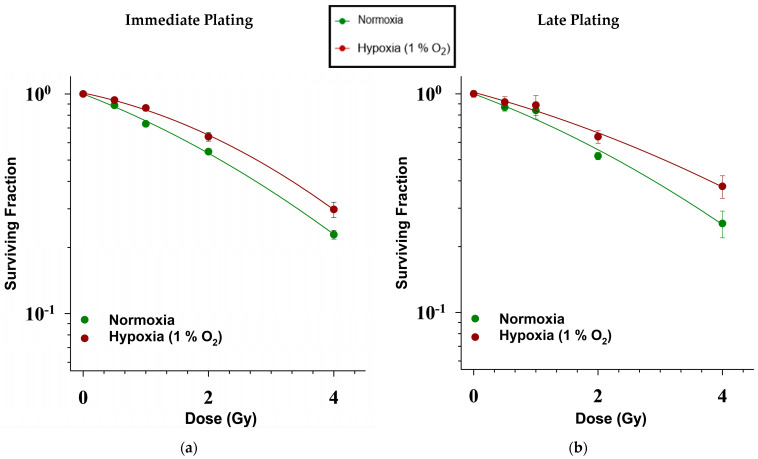
Survival of normoxic and hypoxic H358 cells after exposure to X-rays. The curves depict survival on a semi-logarithmic plot as a function of the dose of X-rays: (**a**) H358 cells were seeded immediately after irradiation—Immediate plating; (**b**) H358 cells were seeded after an incubation period of 24 h following irradiation. *n* = 18. Error bars represent SE.

When incubated under hypoxia, the p53-null H358 cells demonstrated higher cell survival after exposure to X-rays in comparison to their normoxic controls (Figure 2). This enhancement in radioresistance under hypoxia (48 h pre-incubation under 1% O_2_ followed by all subsequent handling under 1% O_2_ as well) was observed in both immediate and late plating experiments.

For immediate plating, the irradiated cells were seeded immediately after irradiation for the growth of colonies. In this case, the shoulder of the survival curves of the chronically hypoxic cells was broadened in comparison to survival under normoxia (Figure 2). This was objectively confirmed by an increase in the quasi-threshold dose Dq under hypoxia compared to normoxia (Table 2). The α/β values for hypoxic cells were also lower than those for normoxic cells, indicating greater radioresistance under hypoxia (Table 3).

For late plating, the irradiated cells were seeded for colony growth 24 h after irradiation. In that case, the survival curve of hypoxic cells is relatively straight and less steep compared to the survival curves of normoxic cells. This was objectively confirmed by a decrease in the survival curve slope (1/D_0_) under hypoxia compared to normoxia (Table 2). As was the case for immediate plating, the α/β values for hypoxic cells were lower than those for normoxic cells, indicating greater radioresistance under hypoxia (Table 3).

Hypoxia-induced radioresistance is greater in late plating experiments compared to immediate plating experiments, as exemplified by the higher D_0_ values and the greater oxygen enhancement ratios (OER) (Table 2).

### 2.3. Hypoxia Does Not Affect Double-Strand Break Formation or Resolution in H358 After X-Ray Exposure

**Figure 3:**
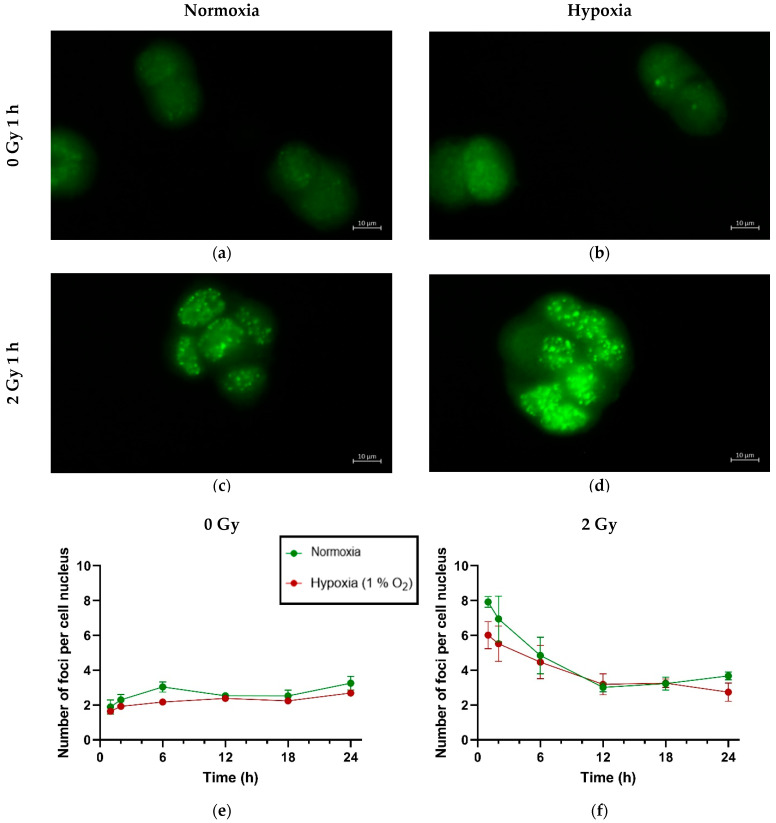
γH2AX foci induction and repair over time in H358 cells under normoxia and chronic hypoxia (1% O_2_) following X-ray exposure. Exemplary images of γH2AX foci (green fluorescence) in H358 cells 1 h after exposure to 0 Gy (**a**,**b**) or 2 Gy (**c**,**d**) X-rays under normoxia (**a**,**c**) and hypoxia (**b**,**d**), scale bar 10 µm. Repair kinetics after mock-irradiation (0 Gy) (**e**) and after exposure to 2 Gy X-rays (**f**). Hypoxic cells were kept under hypoxia until fixation. *n* = 3. Error bars represent SE, standard error.

Although there was a trend of lower γH2AX foci induction in hypoxic H358 cells compared to normoxic controls after an X-rays dose of 2 Gy, this trend was not statistically significant (Figure 3, Table 4). Furthermore, the resolution of the foci indicative of DNA double-strand break (DSB) repair followed essentially similar kinetics over time under both normoxia and hypoxia. No change was observed in the background foci levels under hypoxia compared to normoxia.

### 2.4. Hypoxia Delays and Reduces G2 Arrest in H358 Cells After X-Ray Exposure

**Figure 4:**
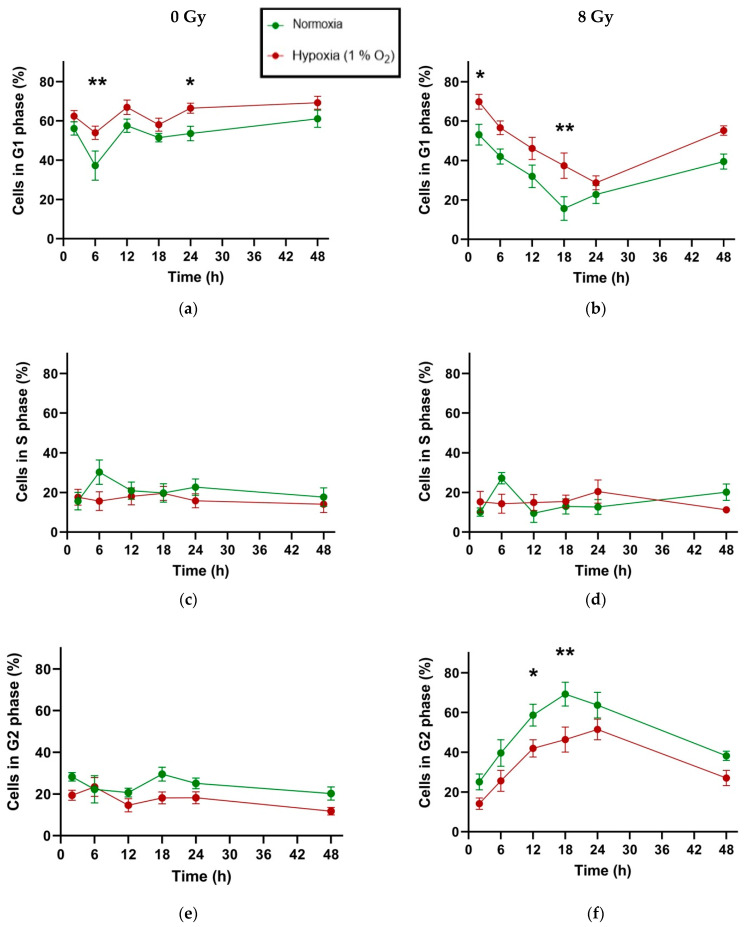
Distribution of H358 cells across different cell cycle phases over time following irradiation (8 Gy X-rays). (**a**) percentage of cells in G1 phase in unirradiated controls under normoxia (green) and hypoxia (red); (**b**) percentage of cells in G1 phase in irradiated cells under normoxia (green) and hypoxia (red); (**c**) percentage of cells in S phase in unirradiated controls under normoxia (green) and hypoxia (red); (**d**) percentage of cells in S phase in irradiated cells under normoxia (green) and hypoxia (red); (**e**) percentage of cells in G2 phase in unirradiated controls under normoxia (green) and hypoxia (red); (**f**) percentage of cells in G2 phase in irradiated cells under normoxia (green) and hypoxia (red). Significant differences in the mean cell populations are represented with asterisks for normoxia vs. chronic hypoxia (1% O_2_); *: *p* < 0.05; **: *p* < 0.01; *n* = 3. Error bars represent SE, standard error.

Chronic hypoxia reduces the percentage of actively cycling H358 cells, as demonstrated by the redistribution of hypoxic cells out of the S and G2 phases into the G1 phase of the cell cycle when compared with normoxic controls (Figure 4). However, this trend does not reach statistical significance in this cell line at 1% O_2_.

When H358 cells were irradiated with 8 Gy of X-rays, there was a strong induction of cell cycle arrest at the G2/M checkpoint in normoxic cells. Under hypoxia, the G2 arrest occurred in a lower proportion of H358 cells, and this difference was statistically significant 12 and 18 h after irradiation. This was complemented by a statistically significant difference in the proportion of cells in G1 under normoxia and hypoxia, whereby a greater number of hypoxic cells were in G1 18 h after X-ray exposure compared to normoxic cells.

Furthermore, the hypoxic cells appeared to be more delayed in their response to X-ray exposure, whereby the maximum G2 arrest occurred 24 h after irradiation as opposed to 18 h after exposure in normoxic controls (Table 5). Parallel to the G2 arrest, the percentage of cells in G1 reached a minimum at the same time points after irradiation. The G2 arrest was largely resolved 48 h after irradiation in both normoxic and hypoxic cells.

Medium change immediately after irradiation appeared to produce an upward spike in the proportion of cells in the S phase under normoxia 6 h after irradiation, which was absent in hypoxic cells.

### 2.5. Earlier Increase in Rate of Cytokines Secretion in Hypoxic H358 Cells Compared to Normoxic Controls

**Figure 5:**
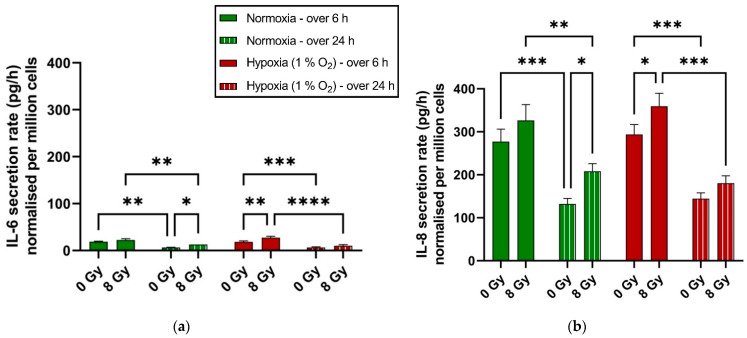
Rate of cytokine secretion (in pg/h per 1 million cells) of normoxic and hypoxic (1% O_2_) H358 cells in the first 6 h and 24 h after X-ray exposure (8 Gy), (**a**) IL-6, (**b**) IL8/CXCL8. *: *p* < 0.05; **: *p* < 0.01; ***: *p* < 0.001; ****: *p* < 0.0001; *n* = 3. The bars represent SE, standard error.

Both hypoxia and X-ray exposure might affect the production and secretion of inflammatory cytokines such as IL-6 and IL-8. Therefore, we compared the secretion rate of irradiated to mock-irradiated cells for both hypoxia and normoxia, and of hypoxic to normoxic cells without and with X-irradiation. In addition, we compared the secretion rate during the first 6 h after irradiation to an overall time period of 24 h after irradiation. In H358 cells, under all experimental conditions, the rate of secretion (in picogram per hour per one million cells—pg/h per 10^6^ cells) of IL-8 was greater than that of IL-6 (Figure 5a,b). Additionally, over 24 h following irradiation or medium change, the rate of cytokine secretion was greater during the first 6 h compared to the entire period.

Regarding both IL-6 (Figure 5a) and IL-8 (Figure 5b), chronic hypoxia did not differentially impact their rate of secretion (in pg/h per 10^6^ cells) as indicated by the no statistically significant difference between secretion rates in normoxic and hypoxic controls (0 Gy) for both time points (6 and 24 h). X-ray exposure (8 Gy) leads to a statistically significant increase in their rate of secretion under hypoxia within the first 6 h, and after 6 h but within the first 24 h under normoxia.

### 2.6. Differential Gene Expression of Irradiated H358 Cells Under Hypoxia

#### 2.6.1. Gene Expression Varies in H358 Under the Influence of Hypoxia and Irradiation

Comparison groups were designed to assess DDR in irradiated normoxic samples (N8) compared to un-irradiated normoxic samples (N0), irradiated hypoxic samples (H8) in comparison to un-irradiated hypoxic samples (H0), un-irradiated hypoxic samples (H0) in comparison to un-irradiated normoxic samples (N0), and irradiated hypoxic samples (H8) in comparison to irradiated normoxic (N8) samples.

The total number of significant differentially expressed genes (DEGs) upregulated or downregulated in each group comparison are depicted in Table 6.

**Figure 6:**
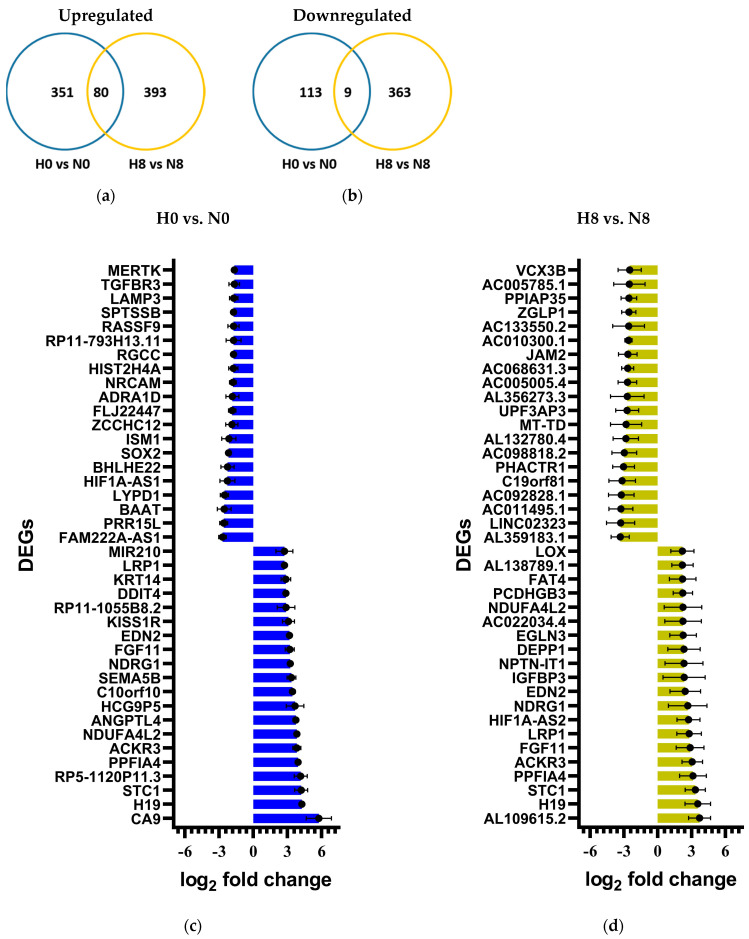
Differential gene expression of hypoxic H358 cells without (blue) or with (yellow) X-ray exposure. Venn diagrams showing overlap of significantly upregulated (**a**) and downregulated (**b**) DEGs observed in hypoxic H358 cells after incubating them under hypoxia (H: 1% O_2_ for 52 h) with or without X-ray exposure (8 Gy) in comparison to normoxic controls (N). Significant DEGs in hypoxic (H) compared to normoxic (N) H358 cells without (H0 vs. N0) (**c**) and with X-ray exposure of 8 Gy (H8 vs. N8) (**d**). Before irradiation, hypoxic cells were incubated with 1% O_2_ for 48 h. Gene expression was analyzed 4 h after irradiation. *n* = 4. The bars represent SE, standard error.

To compare the effect of chronic hypoxia without irradiation (H0 vs. N0) and with irradiation (H8 vs. N8), the number of overlapping DEGs between the two compared groups were evaluated (Figure 6a,b). Individual DEGs (top 20 upregulated and downregulated) are represented in Figure 6c,d.

**Figure 7:**
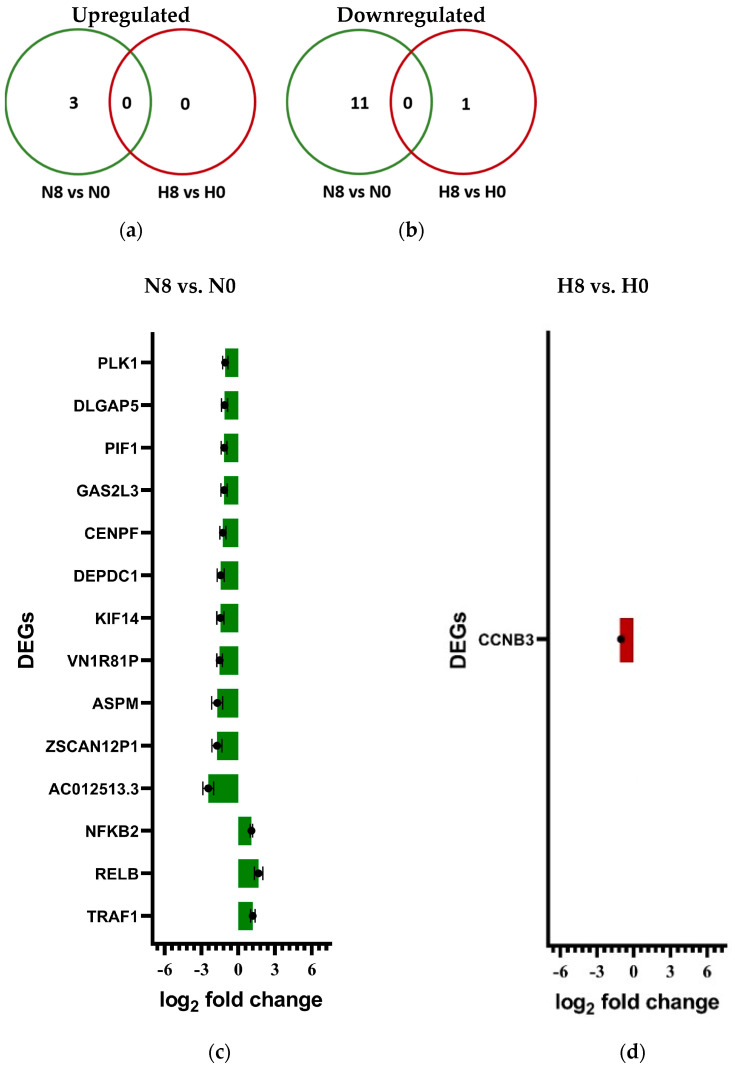
Differential gene expression of X-irradiated H358 cells with incubation under either normoxia (green) or hypoxia (red). Venn diagrams showing overlap of significantly upregulated (**a**) and downregulated (**b**) DEGs following X-ray exposure (8 Gy) of hypoxic (H) and normoxic (N) H358 cells in comparison to unirradiated controls (0 Gy). Significant DEGs in normoxic (N) (**c**) and hypoxic (H) (**d**) H358 cells 4 h after exposure to X-rays (8 Gy). Before irradiation, hypoxic cells were incubated with 1% O_2_ for 48 h. *n* = 4. The bars represent SE, standard error.

To compare the effect of radiation exposure under normoxia (N8 vs. N0) and under hypoxia (H8 vs. H0), the number of overlapping DEGs between the two compared groups were evaluated (Figure 7a,b). Individual DEGs are represented in Figure 7c,d.

#### 2.6.2. Hypoxia Leads to the Differential Expression of DNA Damage Response Genes in H358 Cells

DNA damage response (DDR) was assessed in terms of the differential expression of member genes of the cell cycle (Table 7), DNA damage repair (Table 8), and cell death pathways (Table 9) using the KEGG cellular pathways database.

#### 2.6.3. Hypoxia Leads to the Differential Expression of NF-κB Target Genes in H358

Inflammatory response to radiation was assessed in terms of the differential upregulation of the target genes of NF-κB (Table 10), which is the chief inflammatory mediator in mammalian cells.

## 3. Discussion

In p53-null H358 cells, we observed hypoxia-induced radioresistance which could not be explained by a difference in the DNA DSB induction and repair. This resistance was associated with a decreased X-ray-induced G2/M arrest, a higher proportion of cells in the more radioresistant G1 phase of the cell cycle, and a more rapid upregulation of proinflammatory cytokine secretion under hypoxia.

### 3.1. Clonogenicity of p53-Null H358 Cells Is Greater Under Chronic Hypoxia Compared to Normoxia

Our study demonstrated a statistically significant increase in the colony-forming ability of H358 cells under the influence of 72 h of chronic hypoxia when compared with their normoxic controls (Table 1). The same was not observed in the case of A549 cells, which were used as a reference cell line and demonstrated a statistically significant decline in clonogenicity following hypoxic exposure (72 h). Furthermore, A549 cells grew from a very small number of cells (around 100 per 6 cm Ø Petri dish) into colonies under both normoxia and hypoxia in fresh medium, while the plating efficiency of H358 cells was lower under normoxia. Under hypoxia, H358 cells were incapable of growing from a small starting number into colonies in fresh medium. Therefore, H358 cells were supported in colony growth by adding 50% conditioned medium, as described in Section 4.3. This was performed for both the normoxic and the hypoxic condition. This finding suggests that p53-deficient H358 cells require secreted factors from the conditioned medium, e.g., growth factors [41,42], cytokines, or extracellular matrix components [43], to start proliferation from a very low cell number, while p53-wt A549 are independent of those. Interestingly, it was previously shown that H358 cell secrete high levels of the epidermal growth factor receptor (EGFR) ligand amphiregulin that could induce the autonomous survival of NSCLC cells [44]. In general, the secretome of tumor cells was shown to promote, e.g., invasion and migration [45,46], and it might therefore represent a therapeutic target in cancer treatment.

The different effects of hypoxia on the clonogenicity of H358 and A549 cells may in part be explained by the greater apoptosis and senescence in the p53-wt A549 cells than the p53-null H358 cells, in response to chronic hypoxia. While p53-independent mechanisms for apoptosis have been characterized, the presence of a functional p53 gene is the most important factor for the activation of the intrinsic and also the extrinsic apoptotic pathways [47,48]. Similarly, together with p16, the role of p53 in the activation of senescence, generally characterized by an irreversible cell cycle arrest, in response to stressors such as chronic hypoxia is well characterized [49,50]. However, genetic differences other than the p53 status might exist between H358 and A549 cells, which could be the cause for the different effect of hypoxia on clonogenicity in the two cell lines.

Besides the evasion of cell death pathways, the greater clonogenicity of H358 cells in comparison to A549 cells under chronic hypoxia (1% O_2_) may also be due to a greater stimulation of growth and survival pathways. It is well established that tumor hypoxia may lead to enhanced clonogenicity and “stemness” in some cells through various cellular adaptations, most commonly by increasing the nuclear accumulation of the activated hypoxia-inducible factor 1 (HIF-1), which in turn upregulates the transcription of several genes, inducing stem-cell-like properties within the tumor cells [51,52,53]. Additionally, the p53 inactivation in cancer cells, including those of NSCLC, has been reported to further facilitate HIF-1 heterodimerization, its subsequent nuclear translocation, and thus the HIF-1-induced pro-survival and proliferative processes [35,54].

### 3.2. Radioresistance Increases in p53-Null H358 Cells Under Chronic Hypoxia Compared to Normoxia

The enhanced clonogenicity of H358 cells under chronic hypoxia translated into the greater radioresistance of H358 cells in comparison to normoxic controls following X-ray exposure (Figure 1). Greater radioresistance to X-rays under the influence of chronic hypoxia (0.1–1% O_2_) has been widely reported previously for a variety of cancer cells, including those of NSCLC [17,19,55,56]. However, we did not find any comparative reports regarding the radiosensitivity of p53 null cells, such as H358, under chronic hypoxia. Such information may be important because p53 is important for both cell death and DNA repair pathways; in the absence of a p53-mediated DDR, the probability of cancer cells’ survival may either increase due to the failed activation of cell death pathways, like apoptosis and senescence, or it may decrease due to ineffective DNA repair [57].

Our late plating (LP) experiments (cells’ seeding carried out 24 h after irradiation) revealed a higher OER than immediate plating (IP; cells were seeded immediately after X-ray exposure), thus inducing a greater effect of chronic hypoxia in enhancing radioresistance in the case of LP compared to IP. Cell survival following LP is generally greater than that after IP, as indicated by the higher values of D_0_ (dose needed to reduce survival to 37%) regardless of oxygen concentration (Table 2). Better survival after LP is attributed to the repair of potentially lethal damage (PLD) compared to IP [58]. PLD is defined in radiobiology as DNA damage which can cause cell death, but this is preventable if post-irradiation conditions allow for DNA repair [59]. Thus, higher OER following LP indicates that chronic hypoxia (1% O_2_) is not just conducive to DNA repair in H358 cells but may even be more so than normoxia (20% O_2_). This assumption is further supported by the observation of greater D_q_ values and, hence, survival curve “shouldering” under hypoxia (Table 2), which is usually interpreted as indicating that higher doses are needed to saturate the DNA repair machinery of cells [60].

Table 11 compares our current study with our previous work, employing A549 cells to perform colony-forming ability assays in essentially the same experimental conditions. Hypoxia-induced radioresistance is greater in H358 compared to A549 cells. This is exemplified by the D_0_ and OER values, which are greater for hypoxic H358 than for hypoxic A549 cells when compared to their respective normoxic controls.

Under normoxic conditions, the difference in p53 status may explain the enhanced radioresistance in H358 cells compared to A549 cells. The inactivation of p53 has been reported to reduce radiation-induced cytotoxicity in a variety of prostate cancer cell lines by limiting cell senescence and clonogenicity [61]. The same has also been reported in murine gastrointestinal and fibroblast cells, with the increased radioresistance attributed to the inhibition of p53-dependent apoptosis [62,63]. Whether this explanation can be extrapolated to justify the greater radioresistance in the setting of chronic hypoxia observed in p53 null H358 cells compared to p53wt A549 cells is an open question. The inhibition of HIF-1 in p53-deficient NSCLC cells has been reported to increase radiosensitivity [40]. On the other hand, its activation has been reported to increase radioresistance by reprogramming cellular energy metabolism, promoting an epithelial–mesenchymal transition (EMT), and redistributing cells through the cell cycle [64]. Therefore, it is likely that the combined effect of p53 inactivation and chronic hypoxia may lead to greater radioresistance in p53-null cells, such as H358, compared to their wild-type counterparts.

### 3.3. H358 Cells Exhibit a Comparable Induction and Resolution of X-Rays-Induced DSBs Under Normoxia and Hypsoxia

Following X-ray exposure (2 Gy), a comparison of γH2Ax foci in H358 cells under normoxia and hypoxia (1% O_2_) revealed no statistically significant difference in foci induction and resolution between the two oxygen conditions. However, there was a trend in favor of lower γH2Ax foci induction under hypoxia in our experiments with exposure to 2 Gy X-rays (Figure 2b, 1 and 2 h time points). Recently, hypoxia-induced radioresistance in three NSCLC cell lines (A549, H460 and Calu-1) has been reported to be associated with less γH2Ax foci formation after X-ray exposure than in normoxic controls. The study demonstrated that a decline in the production of reactive oxygen species (ROS) under hypoxia led to a smaller number of DSBs following irradiation [19]. However, this study compared 0.1% O_2_ with normoxic conditions, which may be responsible for the measurable difference in γH2Ax foci induction. Furthermore, the study reported a smaller number of DSBs in hypoxic cells in comparison to normoxic controls only at higher doses (≥4 Gy). Indeed an O_2_ concentration of 0.1% has been reported to affect radiosensitivity far more negatively than that of 1%, as a result of significantly reducing ROS production [65,66]. On the other hand, enhanced radioresistance due to chronic exposure to 1% O_2_, as observed in our cell survival studies may be the result of cellular adaptations rather than reduced DSB induction under hypoxia compared to normoxia. Our findings are supported by similar studies evaluating γH2Ax foci induction under hypoxia in SQ20B and FaDu, which are laryngeal and hypopharyngeal squamous cell carcinoma cell lines, as well as in A549, which is an NSCLC cell line [17,67]. These studies report no statistically significant difference in γH2Ax foci induction or resolution between the two oxygen conditions (1% and 21% O_2_). Of note is also the appearance of relatively few γH2Ax foci following a 2 Gy dose in H358 regardless of the oxygen concentration in our study. To date, no other publication evaluating γH2Ax foci induction by ionizing radiation is available for this cell line. However, observing γH2Ax foci in the numerical range as reported in our work is not uncommon and may indeed occur, depending on the inherent radiosensitivity differences among various cell lines as well as the choice of magnification and the microscopic technique [68].

### 3.4. G2/M Redistribution Following X-Ray Exposure in H358 Cells Is Reduced and Delayed Under Chronic Hypoxia

A radiation-induced G2 arrest might be affected by hypoxia-induced G1 arrest, which results in a lower number of cells which cycle actively. Therefore, we evaluated the cell cycle distribution of chronically hypoxic, unirradiated cells. Under the influence of chronic hypoxia (1% O_2_), unirradiated H358 cells demonstrated no statistically significant increase in cells in the G1 phase, except at the 24 h time point (Figure 3a). This is noteworthy because, generally, such a hypoxic environment is reported to lead to G1 arrest in cells most affected by low oxygen concentrations due to reduced growth signaling [64]. One mechanism reported to explain this phenomenon in the NSCLC cell line A549 involved hypoxia-induced HIF-1α to negatively regulate Cyclin D1, thereby decreasing the number of cycling cells, which led to an increase in quiescent cells in the G1 phase [69]. Chronic hypoxia can also directly activate the G1/S checkpoint in a p53-dependent fashion [70]. We previously reported that the proliferation rate of NSCLC cells (A549and H358) under chronic exposure to 1% O_2_ declined compared to normoxic controls, but unlike the A549 cells, this did not translate into a greater G1 fraction in H358 cells across multiple time points [28]. We postulated no significant impact of hypoxia on the G1 fraction in H358 cells compared to A549 cells due to their p53 null status, as p53 is essential for cell cycle arrest at the G1/S checkpoint. The failure of hypoxic H358 cells to significantly redistribute toward the G1 phase at 1% O_2_ in our study rules out the possibility of their enhanced radioresistance being attributed to a greater number of these cells being in G1 at the time of irradiation, as cells are most radioresistant when they are noncycling and quiescent in the G1 phase [64,71].

Following an X-ray exposure of 8 Gy, the H358 cells demonstrated a G2 cell cycle arrest (Figure 3f). The G2/M checkpoint is not solely reliant on p53 and can be activated through alternative signal transducers such as CHK1, Plk1, and WEE1 [72,73]. Previously, H358 cells have been reported to undergo G2 arrest in response to irradiation in a dose-dependent manner, peaking at around 24 h [74]. In our study, the peak G2 cell fraction was significantly lower under hypoxia compared to normoxia (Table 5). This is contrary to the findings previously reported for human osteosarcoma cells, the U2OS when maintained at ≤0.2% O_2_ and subsequently irradiated with low doses of X-rays (≤1 Gy) [75]. The study cites the reduced cyclin B expression as a potential mechanism. However, in A549 cells maintained at 1% O_2_ and subsequently irradiated with 8 Gy, as was the case in our study, the percentage of cells exhibiting a G2 block was lower compared to normoxic controls [17]. This study also reports no differential regulation of cyclin B under hypoxia (4 h) after irradiation in A549 cells. The deterioration of the G2 arrest under the hypoxic environment of 1% O_2_ has also been validated in SKOV3 human ovarian cancer cells following treatment with Paclitaxel, postulating the inhibition of the Src/Stat3/HIF-1α pathway as a potential mechanism [76].

Furthermore, in our study, hypoxic cells attained the maximum G2 fraction at a later time point (24 h) compared to normoxic controls (18 h). This is most likely an effect of the slower proliferation rate under hypoxia compared to normoxia [28].

Most importantly, our cell cycle analysis revealed that the G2 fraction in irradiated normoxic H358 cells was significantly greater than the unirradiated normoxic controls even after 48 h, which was not the case for hypoxic cells. This alludes to the possibility of more H358 cells becoming senescent following X-ray exposure under normoxia compared to hypoxia. The ability of H358 cells to evade senescence and continue cycling in greater numbers under hypoxia compared to normoxia after X-ray irradiation may explain their enhanced radioresistance when maintained for prolonged periods at 1% O_2_. Radiation-induced senescence is well characterized in numerous cancer cells, including NSCLC cells such as A549 and H460, by p53-dependent mechanisms [77,78,79]. While there are reports of relatively reduced senescence in p53 null cells [80], mechanisms independent of functional p53 also exist for inducing cell senescence, such as p16 induction or transformation/transcription domain-associated protein (TRRAP) depletion [49,81]. The effect of hypoxia in inducing senescence is much more nuanced compared to ionizing radiation, with reports that hypoxia can both induce and inhibit it, depending on its severity, duration, as well as the cell type being studied [82,83]. Whether the combined effect of irradiation and hypoxia is a relative suppressor of senescence in the p53 null H358 cells would require further investigation using senescence-specific biomarkers.

### 3.5. Chronic Hypoxia Accelerates the Pro-Survival Inflammatory Response to X-Ray Exposure in p53-Null H358 Cells

The role of the hypoxia-triggered inflammatory response in tumor propagation and treatment resistance is well characterized [84,85]. We studied the hypoxia-induced inflammatory response in H358 cells by evaluating IL-6 and IL-8 secretion, as both proinflammatory cytokines have been reported to positively impact the cell proliferation, immune evasion, angiogenesis, and metastatic potential in NSCLC [86,87,88].

H358 cells exhibited a several-fold higher secretion of IL-8 compared to IL-6 in our study, regardless of oxygen concentration or irradiation. Our previous work comparing IL-6 and IL-8 secretion in A549 cells under a similar experimental set-up did not elucidate such a difference in the case of A549 [29]. However, another study comparing IL-8 secretion in A549 and H358 cells reported IL-8 secretion values per 10^6^ cells in the same range, for both H358 and A549, as reported in our current and previous work, respectively [89].

Time had the greatest impact on the secretion rate of both cytokines, as it was always greater in the first 6 h compared to the rate over the entire 24 h period, regardless of oxygenation or irradiation status. However, this is probably best explained by the medium change at the beginning of the experiment (time = 0 h) and the subsequent negative feedback loops coming into play, as the cytokine concentration in the medium increased over time.

**Figure A1:**
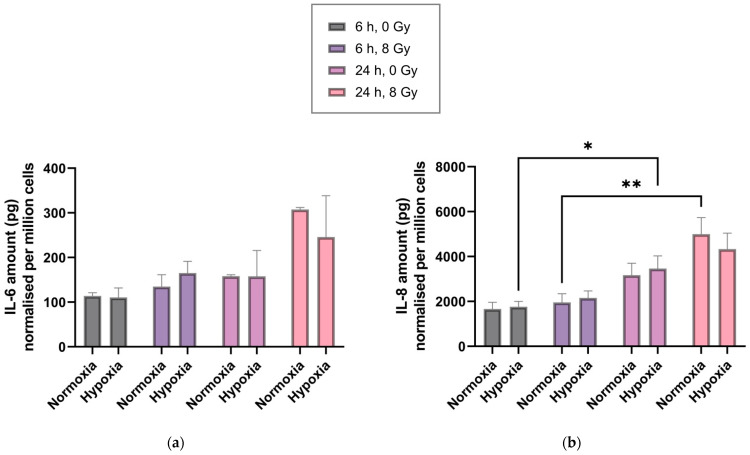
Total amount of cytokine secretion (in pg per 1 million cells) of normoxic and hypoxic (1% O_2_) H358 cells in the first 6 h and 24 h after X-ray exposure (8 Gy), (**a**) IL-6, (**b**) IL8/CXCL8. *n* = 3. The bars represent SE, standard error. *: *p* < 0.05; **: *p* < 0.01.

Hypoxia (1% O_2_) in the absence of irradiation did not affect the cytokine secretion rate in H358 cells. Irradiation increased the secretion rate of both cytokines within the first 6 h in hypoxic cells in a statistically significant manner, which was not the case in normoxic cells. However, X-ray exposure increased the cytokine secretion rate in normoxic H358 cells over the entire course of the 24 h. We were able to confirm through further analyzing the data (Figure A1 in the Appendix A) that there was no statistically significant difference in the total amount (pg) of secreted cytokines between normoxia and hypoxia, with or without irradiation. This is partially contrary to our previous work employing A549 cells under a similar experimental set-up, which demonstrated an overall increase in the total amount of IL-8 but not IL-6 that was secreted under hypoxia as well as that secreted following irradiation under hypoxia, when compared to the corresponding normoxic controls [29]. It remains to be evaluated whether the p53 null status of H358 cells can explain this difference in A549 cells. It is generally understood that p53 acts antagonistically to NF-κB to suppress the expression of inflammatory cytokines. Therefore, loss of the p53 function can enhance the potential for cytokine secretion regardless of the oxygenation status [90]. It has been previously shown that NF-κB is spontaneously activated in H358 cells [91]. Whether this masks the positive effect of hypoxia on cytokine secretion relative to normoxia would require further investigation.

### 3.6. Gene Expression After X-Ray Exposure Under Chronic Hypoxia in p53-Null H358 Cells Favors Cell Survival Through the Epithelial–Mesenchymal Transition

The gene expression data confirmed the results of earlier functional experiments and provided some new information that can direct future studies.

The expression of cell cycle genes (Table 7) under the combined effect of chronic hypoxia and X-ray exposure highlighted the relative regulation of genes inducing G2/M phase transition, namely, the growth arrest and DNA damage inducible gamma (GADD45G) gene and the protein kinase, DNA-activated, catalytic subunit (PRKDC) gene. The downregulation of GADD45G and upregulation of PRKDC in hypoxic H358 cells following X-ray exposure may explain why fewer hypoxic cells underwent G2 arrest following irradiation, compared to the normoxic controls in our cell cycle studies. GADD45G is a key mediator of the G2/M checkpoint [92,93]. It is reported to be downregulated in NSCLC compared to normal lung tissue, and its downregulation is associated with poorer treatment outcomes in Hepatocellular carcinoma [94,95]. Similarly, PRKDC upregulation has been reported to induce a G2/M transition in MCF-7 breast cancer cells [96] and the inhibition of its protein product has been reported to induce a G2/M arrest in primary NSCLC cells [97]. Additionally, the strong downregulation of cyclin-dependent kinase inhibitor 1C (CDKN1C) under the effect of hypoxia, and even more so following X-ray exposure under hypoxia, was observed in H358 cells, which may facilitate both G1/S and G2/M transitions [98]. Interestingly, CDKN1C under-expression is also associated with pluripotency and tumorigenesis in a variety of cancers such as breast, gastric, pancreatic, and urothelial carcinomas [99,100].

Only a few DNA repair genes were significantly differentially expressed in hypoxic and X-irradiated H358 cells; the expression of two polymerases (DNA polymerase epsilon 4-POLE4 and DNA polymerase delta 2-POLD2) was reduced (Table 8). POLD2 is, among others, involved in the DNA mismatch repair and corrects mismatched DNA bases arising from multiple sources, including polymerase errors and base damage, especially oxygen-induced mismatches [101], which might be reduced under hypoxia. PRKDC, which encodes the catalytic subunit of the DNA-dependent protein kinase (DNA-PK), was slightly upregulated in X-ray-irradiated hypoxic cells; as this was the only gene of the non-homologous end-joining DNA DSB repair pathway that was differentially regulated, the biological relevance of this finding might be low. X-ray irradiation alone did not significantly change the expression of DNA repair pathway genes in H358 cells. This might be explained by the absence of a p53 response.

Many cell death-promoting genes were upregulated following the irradiation of chronically hypoxic H358 cells (Table 9). Most of these genes induce a cellular senescence and apoptosis in the presence of functional TP53 cells [102,103,104,105,106,107]. Their relative overexpression may be of little consequence in H358 cells due to the absence of p53. On the other hand, the differential under-expression of IRF3 and IRF7 in hypoxic H358 cells after X-ray exposure may have a protective role in these cells against senescence [108,109]. Lastly, many cell death-promoting genes upregulated under hypoxia following X-ray exposure are also described in the literature to promote an epithelial–mesenchymal transition (EMT) that may increase the likelihood of cell survival [110,111,112,113,114], as observed in our cell survival studies in H358 cells following X-ray exposure under chronic hypoxia. Interestingly, the serpin family E member 1 (SERPINE1), which was upregulated in H358 cells in response to hypoxia, was recently identified as a key hypoxia-related gene and an independent prognostic indicator in breast cancer [115].

A wide variety of NF-κB target genes (Table 10) was upregulated under the effect of chronic hypoxia alone (H0 vs. N0) and under the combined effect of hypoxia and X-ray exposure (H8 vs. N8). Most of these genes are involved in the inflammatory responses enhancing cell survival through the induction of an EMT [116,117,118]. The inflammatory response of H358 cells to chronic hypoxia, especially after X-ray exposure, appears to be a major contributor to their better clonogenic potential and survival compared to normoxic controls.

The TP53 gene in the H358 cell line from the American Type Culture Collection (ATCC) used in this work has undergone complete homozygous deletion [119]. For this reason, the p53 gene was not a part of the RNA library prepared for RNA expression. The expression of the target genes of the transcription factor p53, as listed in the KEGG database (hsa04115), was evaluated during RNA sequencing analysis. The results were not directly related to this work but are shared in the Appendix A (Table A1).

## 4. Materials and Methods

### 4.1. Cell Lines and Culture

H358 cells (human, male, lung adenocarcinoma, p53 null, KRAS-mutated) and A549 cells (human, male, lung adenocarcinoma, p53 wildtype—wt, KRAS-mutated) were obtained from LGC Genomics (Berlin, Germany) [120].

They were routinely cultured in 25 cm^2^ and 80 cm^2^ cell culture flasks (LABsolute, Th. Geyer GmbH, Renningen, Germany) with alpha-minimally essential medium (α-MEM; PAN Biotech, Aidenbach, Germany) containing 10% (*v*/*v*) dialyzed fetal bovine serum (FBS; PAN Biotech), 2% (*v*/*v*) sterile glucose solution (0.94 mol/L), 1% (*v*/*v*) penicillin (10,000 U/mL)/streptomycin (10 mg/mL) (PAN Biotech), 1% (*v*/*v*) neomycin/bacitracin (Biochrom AG, Berlin, Germany), and 1% (*v*/*v*) amphotericin (250 µg/mL) (PAN Biotech). Owing to differences in growth kinetics, H358 cells were cultured at a seeding density of 20,000 per cm^2^, while a seeding density of 5000 per cm^2^ was used for A549 cells in order to ensure 30–40% confluence 48 h after seeding. The cells were regularly tested for mycoplasma contamination by a polymerase chain reaction of supernatants at the Leibniz-Institut DSMZ-Deutsche Sammlung von Mikroorganismen und Zellkulturen GmbH (Braunschweig, Germany) to ensure that they were mycoplasma-free.

The cells were incubated at 37 °C and saturated humidity, either under normoxia (20% O_2_) in a CO_2_ incubator (5% CO_2_; Heraeus HERAcell 150, Thermo Fisher Scientific, Karlsruhe, Germany) or under hypoxia (1% O_2_) in an InvivO_2_ 400 hypoxia workstation (Baker Ruskinn, South Wales, UK) flushed with 5% CO_2_, 1% O_2_, and 94% N_2_. The incubation time in the culture under normoxia or hypoxia before irradiation was 48 h to allow cells to enter the exponential growth phase. The medium change, fixation, or lysis of hypoxic cells were performed in the hypoxia workstation. The medium and reagents used for the purpose were degassed by warming them to 25 °C in the Sonorex Digiplus ultrasonic water bath (Bandelin, Berlin, Germany), at an ultrasound frequency of 35 kHz for 40 min, followed by placing them in the hypoxia workstation for another 40 min with loosened bottle caps before use.

### 4.2. Irradiation

After 48 h of incubation, the H358 cells were irradiated with X-rays (Figure 1). Before transferring the culture flasks for irradiation, their caps were tightened. The flasks containing hypoxic cells were shifted in air-tight boxes before exporting them out of the hypoxia workstation through the airlock for irradiation. They were only taken out from the air-tight boxes for the brief minutes of actual irradiation. Immediately afterwards, the flasks were returned to the air-tight boxes and transported quickly back to the hypoxia workstation. Before the actual experiments, oxygen was determined several times using the Seven2go dissolved oxygen meter S9 (Mettler Toledo, Giessen, Germany) to ensure that this method did not lead to any significant change in oxygen concentration in the medium inside the flasks housing the hypoxic cells.

X-ray exposure (voltage: 200 KV; current: 15 mA; LET: 0.3–3.0 KeV/µm) was performed in an RS 225 X-ray chamber (X-strahl, Ratingen, Germany) at the Institute of Aerospace Medicine, DLR, Germany. The dose rate was adjusted to 1.0 Gy/min by setting the distance of the sample to the exit window of the X-ray source to 450 mm. A copper (Cu) filter with a thickness of 0.5 mm placed at the exit window eliminated low-energy X-rays. Depending on the specific experiments performed, cells were irradiated either in cell culture dishes (Ø 3 cm or 6 cm) or in cell culture flasks (25 cm^2^ or 80 cm^2^). During each irradiation, the dose rate and the accumulated dose were monitored using the ionization chamber TM30013 connected to UNIDOS^webline^ dosimeter (PTW, Freiburg, Germany).

After irradiation, a medium change was performed and the cells were then incubated further, either at 20% O_2_ (normoxia) or 1% O_2_ (hypoxia), for variable time periods, depending on the specific experiments (Figure 1).

### 4.3. Cell Survival Analysis Following X-Ray Exposure Under Normoxia and Hypoxia

Puck‘s colony-forming ability (CFA) assay was performed to compare surviving cell fractions of H358 cells cultured under normoxia (20% O_2_) and hypoxia (1% O_2_) following different doses of X-rays (0, 0.5, 1, 2, and 4 Gy). The mock-irradiated samples (0 Gy) were additionally used to compare plating efficiency under normoxia and hypoxia (Figure 1a).

Cells were seeded in 25 cm^2^ flasks and preincubated under normoxia or hypoxia for 48 h. Cells were irradiated as described in Section 4.2. The irradiated and mock-irradiated cells were trypsinized and seeded in Petri dishes (Ø 6 cm LABsolute, Th. Geyer GmbH, Germany), either immediately after irradiation (immediate plating) or after a delay of 24 h (late plating). Based on the plating efficiency and the anticipated killing effect of X-rays, the number of seeded cells was adjusted to result in around 75 colonies. The culture medium used to seed cells after irradiation was conditioned (1:1) for colony growth in both normoxia and hypoxia. The medium was conditioned by the incubation of H358 cells (starting cell number of 20,000 cells/cm^2^) in 75 cm^2^ flasks (with a 20 mL medium) for 5 days under normoxia. The medium supernatant was collected, filtered (PES-membrane: 0.2 µm), and stored at 4 °C until use. This is because H358 cells did not grow into colonies at 1% O_2_ when seeded in low numbers, regardless of whether they were irradiated or not.

The cell colonies, once visible, were fixed and stained with 5 mL of crystal violet (0.2% *w*/*v*)—formaldehyde (3.5%)—staining solution per Petri dish for 20 min after removing the culture medium from the Petri dishes. Stained colonies comprising over 50 cells were counted using a manual colony counter (Schuett count, Schuett-biotec, Göttingen, Germany). Survival fractions were determined by dividing the colony count by the number of cells that were seeded for each dose (0, 0.5, 1, 2, and 4 Gy). The 0 Gy samples yielded the plating efficiency of H358 cells grown under normoxia or hypoxia for 48 h (immediate plating) or 72 h (late plating). Survival curves were generated for each oxygen condition and radiation quality by plotting the surviving fractions on a logarithmic scale as a function of the dose on a linear scale. The single-hit multi-target model as well as the Linear Quadratic Model were used to perform the regression analysis of the experimental data, and model parameters, such as D_0_, D_q_, n as well as α and β values were computed [121].

The Oxygen Enhancement Ratio (OER) of hypoxia was calculated by Equation (1), as follows:

(1) $$OER=\frac{D_{0(Hypoxia)}}{D_{0(Normoxia)}}$$

### 4.4. Analysis of DNA Double-Strand Break Induction and Resolution Following X-Ray Exposure Under Normoxia and Hypoxia

DNA double-strand breaks were analyzed through the γH2AX immunofluorescence microscopy of cells grown under normoxia and hypoxia following irradiation with an X-rays dose of 2 Gy (Figure 1b). The cells were fixed in 3.5% formaldehyde for 30 min at 4 °C at various time points (1, 2, 6, 12, 18 and 24 h) after irradiation. The fixed cells were permeabilized by adding a solution of 5% normal goat serum (NGS), 1% dimethyl sulfoxide (DMSO), and 0.3% Triton X-100 in PBS for 1 h at room temperature. They were then stained with the primary antibody, Alexa Fluor 488 Mouse anti-γH2AX clone 2F3 (Biolegend, San Diego, CA, USA), diluted (1:250) in a staining solution comprising PBS with 1% DMSO and 0.3% Triton X-100, and incubated overnight at 4 °C. The next day, after washing three times with PBS, the cells were stained with the secondary antibody, goat anti mouse IgG-Atto488 (1:1000, Sigma Aldrich, Saint Louis, MO, USA), and the nuclear stain 4′,6-diamidino-2-phenylindole (DAPI) (0.5 µg/mL stock solution diluted at 1:400) followed by an incubation of 45 min in the dark at room temperature prior to slide preparation.

Microscopy was carried out using the Zeiss Axio Imager M2 (Carl Zeiss NTS GmbH, Oberkochen, Germany). Eighteen images per cover slip were taken using the DAPI and Atto488 channels, keeping exposure time constant across each biological replicate. The number of γH2AX foci within each cell nucleus was counted using Image J software (version 1.54, National Institutes of Health, Bethesda, MD, USA) [122].

### 4.5. Analysis of Cell Cycle Response Following X-Ray Exposure Under Normoxia and Hypoxia

H358 cells were cultured in Petri dishes (Ø 6 cm) with a seeding density of 20,000 cells/cm^2^ and then incubated for 48 h at either 1% or 20% O_2_. The cells were then irradiated with X-rays (8 Gy) and then re-incubated, all the while maintaining the initial oxygen concentration in their environment (Figure 1b). Over the next 48 h, the cells were detached with a trypsin/EDTA solution (1 mL) at various time points and fixed in 3.5% formaldehyde. At 30 min after fixation, the cells were washed with PBS and the cell nuclei were stained with a 4′,6-diamidino-2-phenylindole (DAPI) solution (500 ng/mL) and Triton X (3 µg/mL) in PBS. The stained cells were incubated for 30 min in the dark at room temperature. The nuclear DNA content of the cells was measured by flow cytometry (Cytoflex S, Beckman Coulter, Indianapolis, IN, USA). This allowed for the determination of the cell cycle phase distribution of the cells following X-ray exposure under hypoxia and normoxia. Excitation of DAPI within cell nuclei was carried out through the violet laser (405 nm), and the resultant blue fluorescence was measured in the fluorescence channel PB450 of the flow cytometer. Gating was carried out in a forward vs. side scatter plot and a PB450 width vs. area plot, and the blue fluorescence histograms of single cells were analyzed by FloJo software (Version 10, BD Biosciences, San Jose, CA, USA) using the Dean-Jett-Fox cell cycle mathematical model available within FloJo [123].

### 4.6. Quantification of Cytokines Secretion Following X-Ray Exposure Under Normoxia and Hypoxia

ELISA kits (Invitrogen, Thermo Fisher Scientific, Karlsruhe, Germany) were used for the quantification of IL-6 and IL-8 in supernatants of H358 cells collected under normoxia and hypoxia, with and without X-ray irradiation (Figure 1b).

Sample supernatants (3 mL) were collected at 6 and 24 h in Eppendorf tubes and stored at −80 °C until subsequent handling. H358 cells in each sample were counted with the LUNA automated cell counter after detaching them with a trypsin/EDTA solution (3 mL). The cell counts were used for the normalization of cytokine production to the cell number.

The primary capture antibodies (100 μL per well; diluted 1:250 in PBS) provided with the kit were used to coat ninety-six-well plates (Corning^TM^ Costar^TM^ 9018 ELISA plate, Kaiserslautern, Germany). The plates were incubated at 4 °C overnight, after which the wells were blocked for nonspecific antibody binding using diluent (200 μL per well; diluted 1:5 in deionized water). The plate wells were then loaded with the samples (100 µL per well), as well as several different known dilutions of the provided standard solution. Following an overnight incubation at 4 °C, the detection antibody (100 µL per well; diluted 1:250 in PBS) provided with the kit was added to the wells. This was followed by a 1 h incubation at room temperature after which Streptavidin-HRP (100 µL per well; diluted 1:100) was added for the IL-6 detection, or Avidin-HRP (100 µL per well; diluted 1:250) was added for the IL-8 detection. The well plates were then incubated at room temperature for 30 min, after which the 3,3′,5,5′-Tetramethylbenzidine (TMB) substrate (100 µL per well) provided with the kit was added to initiate the enzyme reaction which was allowed to continue for 15 min at room temperature and then stopped by the addition of H_2_SO_4_ (100 µL per well; 2N). The incubation steps were carried out on a shaker with 5 washings before and after using a wash buffer (PBS with 0.05% Tween).

### 4.7. Gene Expression Analysis Following X-Ray Exposure Under Normoxia and Hypoxia

To determine the global transcription profile of cells irradiated under normoxia and hypoxia with 8 Gy of X-rays, the culture medium was completely removed 4 h after irradiation (Figure 1b), and the cells were lysed using RLT buffer (Qiagen, Hilden, Germany) containing β-mercaptoethanol (1:100, Sigma Aldrich, St. Louis, MO, USA). RNA was isolated with the RNeasy Mini Kit (Qiagen). RNA concentration and integrity were determined using the RNA 6000 Nano Assay (Agilent Technologies, Böblingen, Germany) in the Bioanalyzer (Agilent Technologies). Then, 3 µg of the total RNA per sample (4 biological replicates per condition), with RNA Integrity Numbers (RIN) above 9.0 were placed on dry ice and sent to GENEWIZ (Leipzig, Germany) for mRNA sequencing in the same run after the Poly (A) selection, using the Illumina NovaSeq6000 (Illumina, San Diego, CA, USA) platform (configuration: 2 × 150 bp, 350 M read pairs). GENEWIZ (Azenta Life Sciences Genomics, Leipzig, Germany) mapped the reads onto the *Homo sapiens* GRCh38 reference genome and calculated the unique gene hit counts falling within the exon regions. Then, the DESeq2 package in R [124] was utilized for the differential gene expression analysis. Gene Set Enrichment Analysis (GSEA) was performed using the expression data [125]. Genes with an adjusted *p*-value < 0.05 and absolute log_2_ fold change > 1 were considered as differentially expressed genes for each group comparison.

### 4.8. Statistical Analysis

Three independent biological experiments with three technical replicates for each experimental condition were conducted for the experiments described in Section 4.3, Section 4.4, Section 4.5, Section 4.6 and Section 4.7, with the exception of the cell survival experiments (three independent biological experiments with six technical replicates each) and the gene expression studies where four independent biological experiments were conducted. Arithmetical means, standard deviations, and standard errors of means (SE) were calculated using Excel software (version 2016, Microsoft corporation, Redmond, WA, USA). Graphs were plotted and tests of significance were performed using GraphPad Prism 9 (Dotmatics, Boston, MA, USA). Two-way ANOVA was used for testing cell cycle, γH2AX, and cytokines data. Multiple two-way unpaired *t*-tests were employed for evaluating CFA data, including plating efficiency under normoxia and hypoxia. To analyze RNA sequencing data, a Wald test was used for calculating *p*-values and the Benjamani–Hochberg test was utilized for finding the adjusted *p*-values (padj) in case of RNA sequencing data.

## 5. Conclusions

Hypoxia-induced radioresistance is present even in the absence of functional p53. This resistance is related to a pro-survival inflammatory response which potentially manifests through the activation of an epithelial–mesenchymal transition in hypoxic H358 cells following X-ray exposure. Ineffective p53-dependent cell death pathways in H358 cells passively support this process.

## Figures and Tables

**Table 1:** Plating efficiencies of H358 and A549 cells grown under normoxia (20% O_2_) and hypoxia (1% O_2_) after either 48 h (immediate) or 72 h (late) of pre-incubation.

Assay Type	Oxygen Protocol	H358		A549	
		Plating Efficiency (%) (µ ± SE) ^a^	*p* Value ^b^	Plating Efficiency (%) (µ ± SE) ^a^	*p* Value ^b^
Immediate plating	Normoxia	18.0 ± 1.0	0.113	27.0 ± 1.0	0.785
	Hypoxia	21.0 ± 3.0		28.0 ± 2.0	
Lateplating	Normoxia	16.0 ± 3.0	0.000	31.0 ± 2.0	0.000
	Hypoxia	28.0 ± 3.0		22.0 ± 1.0	

**^a^** µ, arithmetical mean, SE, standard error, n higher than 12. **^b^** A *t*-test was used to compare plating efficiencies under normoxia and hypoxia for each cell line. H358 cells grew in conditioned medium, while A549 cells were incubated with fresh medium.

**Table 2:** Parameters of the survival curves of H358 cells grown under normoxia or hypoxia following immediate and late plating after X-irradiation. The parameters were generated by modeling experimental data using the single-hit multi-target model.

Assay Type	Oxygen Protocol	D_0_ * (Gy) µ ± SD	D_q_ (Gy) µ ± SD	*N* µ ± SD	OER µ ± SD
Immediate plating	Normoxia	2.59 ± 0.15	0.25 ± 0.15	1.10 ± 0.06	
	Hypoxia	2.98 ± 0.24	0.48 ± 0.22	1.18 ± 0.07	1.15 ± 0.10
Late plating	Normoxia	2.73 ± 0.24	0.28 ± 0.22	1.11 ± 0.08	
	Hypoxia	3.78 ± 0.29	0.34 ± 0.20	1.09 ± 0.05	1.38 ± 0.16

* D_0_ is the negative reciprocal value of the slope in the exponential region of the survival curve and represents the radiation dose needed to reduce the surviving cell fraction to 37% of its initial value in this region of the curve; Dq is the quasi-threshold dose at which the survival curve stops shouldering and straightens out; *n* is the extrapolation number acquired by extending (in the semi-logarithmic graph) the linear part of the curve to the y-axis and is another means to measure the curve shoulder; OER is the oxygen enhancement ratio calculated by dividing the required dose for a particular biological endpoint under hypoxia compared to that under normoxia. µ, arithmetical mean, SD, standard deviation.

**Table 3:** Parameters of the survival curves of H358 cells incubated under normoxia and hypoxia following immediate and late plating after exposure to X-rays. The parameters were generated by modeling experimental data using the linear quadratic model.

Assay Type	Oxygen Protocol	α (Gy^−1^) ^a^ µ ± SD	β (Gy^−2^) ^a^ µ ± SD	α/β (Gy) ^b^ µ ± SD
Immediate plating	Normoxia	0.25 ± 0.03	0.03 ± 0.01	8.18 ± 0.03
	Hypoxia	0.10 ± 0.01	0.05 ± 0.01	2.06 ± 1.00
Late plating	Normoxia	0.22 ± 0.06	0.03 ± 0.02	7.02 ± 1.52
	Hypoxia	0.14 ± 0.04	0.03 ± 0.02	4.85 ± 1.52

**^a^** α and β are constants calculated for the survival curve using the linear quadratic model. **^b^** The ratio α/β is used as a measure of radiosensitivity in radiation oncology, and it represents the dose at which linear and quadratic components of cell killing induced by ionizing radiation are equal. µ, arithmetical mean, SD, standard deviation.

**Table 4:** Number of γH2AX foci in H358 cells 1 h after X-ray irradiation.

Oxygen Protocol	Number of Foci 1 h After Irradiation µ ± SE
Normoxia	7.9 ± 0.5
Hypoxia	6.0 ± 1.3

**Table 5:** Minimum percentage of H358 cells in the G1 and maximum percentage of H358 cells in the G2 phase of the cell cycle following X-ray exposure (8 Gy) along with time after irradiation at which the minimum or maximum percentage were reached.

	G1		G2	
Oxygen Protocol	Minimum% of Cells (µ ± SE) in G1 Phase After Irradiation ^a^	Time After Irradiation ^a^	Maximum% of Cells (µ ± SE) in G2 Phase After Irradiation ^a^	Time After Irradiation ^a^
Normoxia	15.6 ± 5.2	18 h	69.2 ± 5.2	18 h
Hypoxia	28.7 ± 3.0	24 h	51.5 ± 4.5	24 h

**^a^** Minimum and maximum percentages and time after irradiation are derived from the data shown in Figure 4b,f.

**Table 6:** Number of significant differentially expressed genes (DEGs) in H358 cells with and without X-ray exposure (8 Gy) after maintaining them under normoxic (N) or hypoxic (H) (1% O_2_ for 52 h: 48 h pre-incubation before irradiation, 4 h incubation after exposure) conditions, determined by RNA sequencing.

Effect of	Compared Groups	DEGs	
		Upregulated	Downregulated
(a) Hypoxia	H0 vs. N0 *	431	122
(b) Hypoxia and irradiation with X-rays	H8 vs. N8	473	372
(c) Irradiation with X-rays			
Normoxic cells	N8 vs. N0	3	11
Hypoxic cells	H8 vs. H0	0	1

* Based on principal component analysis, batch correction was applied to the comparison group H0 vs. N0 and two biological repeats of hypoxic samples were compared with four biological repeats of normoxic samples.

**Table 7:** DEGs within each group comparison were evaluated against member genes from the human cell cycle pathway (ID hsa0410), in the Kyoto Encyclopedia of Genes and Genomes (KEGG) database. Log_2_FC of significant DEGs influencing cell cycle regulation are highlighted in bold.

Gene Name	H0 vs. N0	H8 vs. N8	N8 vs. N0	H8 vs. H0
*STAG1*	0.12	**1.16**	−1.14	−0.20
*EP300*	−0.02	**1.15**	−1.02	−0.05
*RBL2*	0.40	**1.18**	−0.78	−0.05
*PRKDC*	−0.25	**1.08**	−0.82	0.04
*GADD45G*	−0.41	**−1.52**	1.19	0.05
*CDKN1C*	**−1.42**	**−1.92**	0.70	0.25
*CCNB3*	0.15	1.17	−1.89	**−1.03**
*PLK1*	−0.35	−0.03	**−1.07**	−0.80

**Table 8:** DEGs within each group comparison were evaluated against member genes from the human DNA repair pathways in the KEGG database. Log2FC of significant DEGs influencing DNA damage repair are highlighted in bold.

Gene Name	Repair Pathway	H0 vs. N0	H8 vs. N8	N8 vs. N0	H8 vs. H0
POLE4	BER (hsa03410), NER (hsa03420)	−0.35	**−1.40**	1.22	0.14
POLD2	BER (hsa03410), MMR (hsa03430), NER (hsa03420), HR (hsa03440)	−0.45	**−1.27**	0.89	0.05
PRKDC	NHEJ (hsa03450)	−0.25	**1.08**	−0.82	0.04

**Table 9:** DEGs within each group comparison were evaluated against member genes from several relevant human cell death pathways in the KEGG database. Log_2_FC of significant DEGs influencing cell death activation are highlighted in bold.

Gene Name	Cell Death Pathway Regulated	H0 vs. N0	H8 vs. N8	N8 vs. N0	H8 vs. H0
*IGFBP3*	Cellular senescence (hsa04218)	**2.55**	**2.34**	−0.44	−0.03
*TGFB2*		0.24	**1.08**	−0.96	−0.10
*TGFBR1*		0.42	**1.05**	−0.59	−0.03
*SERPINE1*		**2.11**	0.84	0.08	−0.33
*RBL2*		0.40	**1.18**	−0.78	−0.05
*EIF4EBP1*		−0.22	**−1.44**	1.29	0.05
*CALML6*		-	**−1.90**	1.53	−0.40
*GADD45G*		−0.41	**−1.52**	1.19	0.05
*CCNB3*		0.15	1.17	−1.89	**−1.03**
*APAF1*	Apoptosis (hsa04210)	**1.02**	**1.67**	−0.87	−0.10
*IL1RAP*		0.56	**1.39**	−0.82	0.06
*TXNIP*	Pyroptosis (hsa04621)	**1.70**	1.39	−0.46	−0.21
*ERBIN*		#N/A	**1.18**	−0.90	−0.09
*TAB3*		0.39	**1.16**	−0.88	−0.12
*PKN2*		0.20	**1.14**	−0.81	−0.07
*CARD16*		−0.71	**−1.28**	0.37	−0.26
*IRF3*		−0.14	**−1.41**	1.15	0.03
*IRF7*		0.14	**−1.03**	1.01	0.09
*FTL*	Ferroptosis (hsa04216)	−0.45	**−1.05**	0.69	0.09
*PLA2G4D*	Necroptosis (hsa04217)	**1.83**	1.04	0.00	−0.03
*HIST3H2A*		**−1.08**	−0.32	0.29	0.20
*TLR4*	Necroptosis (hsa04217), pyroptosis (hsa04621)	**−1.29**	0.07	−0.77	0.09
*RIPK3*		**1.17**	0.28	0.55	−0.01
*ITPR2*	Cellular senescence (hsa04218), pyroptosis (hsa04621)	0.27	**1.09**	−0.85	−0.22
SQSTM1	Cellular senescence (hsa04218), necroptosis (hsa04217)	**−1.02**	−0.74	0.11	0.16

**Table 10:** Upregulated DEGs within each group comparison were evaluated against member genes in the Boston University repository of NF-κB target genes. Log2FC of significant DEGs that are NF-κB target genes are highlighted in bold.

Gene Name	H0 vs. N0	H8 vs. N8	N8 vs. N0	H8 vs. H0
*MYLK*	**1.30**	**1.50**	−0.64	−0.13
*F3*	**1.12**	1.26	−0.69	−0.10
*PTGS2*	**1.17**	**1.59**	−0.33	0.14
*TREM1*	**2.60**	1.32	0.35	0.22
*PDGFB*	**2.19**	0.57	1.07	0.17
*ENO2*	**2.58**	1.06	0.82	0.04
*EGFR*	0.47	**1.53**	−1.01	0.01
*DUSP1*	**1.26**	1.14	−0.34	0.09
*REL*	0.41	1.11	−0.51	−0.05
*REV3L*	0.13	**1.00**	−0.83	−0.11
*PTAFR*	**1.93**	−0.05	1.71	0.28
*SERPINE1*	**2.11**	0.84	0.08	−0.33
*BNIP3*	**1.14**	0.97	−0.27	0.01
*AHCTF1*	0.01	**1.18**	−0.92	−0.07
*SLC6A6*	0.82	**1.11**	−0.52	−0.13
*BLNK*	**1.15**	0.93	−0.34	−0.38
*MMP9*	**1.06**	−1.12	1.89	0.24
*ENG*	**1.03**	−0.05	0.42	−0.25
*RELB*	0.30	−0.65	**1.65**	0.74
*TRAF1*	0.04	−0.28	**1.18**	0.72
*NFKB2*	0.40	−0.19	**1.07**	0.59

**Table 11:** Comparison of D_0_ and oxygen enhancement ratio (OER) between H358 (this study) and A549 cells [17] following X-ray exposure under normoxia and hypoxia.

Assay Type	Oxygen Protocol	H358		A549	
		D_0_ ^a^ (µ ± SD) ^b^	OER (µ ± SD)	D_0_ (µ ± SD)	OER (µ ± SD)
Immediate plating	Normoxia	2.59 ± 0.15	1.15 ± 0.10	2.98 ± 0.20	0.57 ± 0.04
	Hypoxia	2.98 ± 0.24		1.68 ± 0.08	
Late Plating	Normoxia	2.73 ± 0.24	1.38 ± 0.16	2.29 ± 0.13	1.10 ± 0.08
	Hypoxia	3.78 ± 0.29		2.50 ± 0.16	

**^a^** D_0_ is the negative reciprocal value of the slope in the exponential region of the survival curve and represents the radiation dose needed to reduce the surviving cell fraction to 37% of its initial value in this region of the curve. **^b^** µ, arithmetical mean, SE, standard error

## Data Availability

Research data are stored in an institutional repository and will be shared upon request to the corresponding author.

## Appendix A

**Table A1:** Expression of p53 target genes in H358 cells under hypoxia (mock-irradiated cells: H0 vs. N0; cells exposed to 8 Gy X-rays H8 vs. N8), and after X-irradiation (8 Gy) under normoxia (N8 vs. N0) or hypoxia (H8 vs. H0). DEGs in H358 cells within each group comparison (Section 2.6.1) were evaluated using the p53 target genes listed in KEGG’s database (KEGG ID: hsa04115). Log2FC values highlighted in bold are significant. ‘NA’ represents no gene expression data.

Gene Name	H0 vs. N0	H8 vs. N8	N8 vs. N0	H8 vs. H0
*APAF1*	**1.02**	**2.30**	**1.30**	0.02
*ATM*	0.09	**1.23**	**1.03**	−0.11
*ATR*	−0.11	**0.97**	**1.00**	−0.09
*BAX*	−0.32	**−0.97**	**−0.64**	0.01
*BID*	−0.30	**−0.47**	−0.23	−0.06
*CCNB1*	**−0.39**	−0.24	0.83	0.69
*CCNB3*	0.15	0.56	**1.52**	**1.10**
*CCND3*	**−0.67**	**−0.99**	**−0.39**	−0.07
*CCNE2*	−0.27	**1.07**	**1.22**	−0.12
*CCNG2*	**1.11**	**2.19**	**1.09**	0.02
*CDK4*	**−0.41**	**−0.71**	**−0.35**	−0.05
*CDK6*	−0.01	**1.23**	**1.18**	−0.07
*CDKN1A*	0.02	**−0.45**	**−0.68**	−0.22
*CDKN2A*	**−0.37**	**−0.64**	**−0.38**	−0.11
*CHEK1*	−0.18	0.20	**0.34**	−0.04
*CYCS*	−0.36	**−0.33**	0.02	−0.01
*DDB2*	0.31	−0.29	**−0.50**	0.09
*EI24*	−0.11	−0.32	**−0.36**	−0.14
*GADD45G*	−0.41	**−1.20**	**−1.01**	NA
*GTSE1*	−0.01	**0.44**	**1.12**	**0.67**
*IGFBP3*	**2.55**	**3.00**	0.51	0.06
*MDM2*	0.22	**0.99**	**0.81**	0.04
*MDM4*	0.18	**0.82**	**0.81**	0.17
*PIDD1*	0.15	−0.48	**−0.71**	−0.08
*PMAIP1*	**−0.73**	−0.03	**0.71**	0.00
*PPM1D*	**−0.43**	0.20	**0.61**	−0.02
*RPRM*	**−1.30**	**−1.86**	−0.09	NA
*RRM2B*	0.23	**1.06**	**0.70**	−0.14
*SERPINE1*	**2.11**	**1.45**	−0.32	NA
*SESN2*	−0.58	**−0.74**	−0.22	−0.07
*SESN3*	−0.17	**0.96**	**0.89**	−0.24
*SFN*	0.14	−0.53	**−0.72**	−0.05
*SHISA5*	−0.26	**−0.73**	**−0.48**	−0.01
*THBS1*	**0.36**	**2.33**	**2.15**	0.18
*TP53I3*	0.40	−0.04	**−0.40**	0.05
